# A Study of 3CLpros as Promising Targets against SARS-CoV and SARS-CoV-2

**DOI:** 10.3390/microorganisms9040756

**Published:** 2021-04-03

**Authors:** Seri Jo, Suwon Kim, Jahyun Yoo, Mi-Sun Kim, Dong Hae Shin

**Affiliations:** Graduate School of Pharmaceutical Sciences, Ewha Womans University, 52, Ewhayeodae-gil, Seoul 03760, Korea; seri9388@gmail.com (S.J.); suwon910228@naver.com (S.K.); noonio@naver.com (J.Y.); shfwk31@ewha.ac.kr (M.-S.K.)

**Keywords:** SARS-CoV-2 3CL protease, drug repurposing, antiviral, FRET, inhibitory compounds

## Abstract

The outbreak of coronavirus disease 2019 (COVID-19), caused by severe acute respiratory syndrome coronavirus 2 (SARS-CoV-2), results in serious chaos all over the world. In addition to the available vaccines, the development of treatments to cure COVID-19 should be done quickly. One of the fastest strategies is to use a drug-repurposing approach. To provide COVID-19 patients with useful information about medicines currently being used in clinical trials, twenty-four compounds, including antiviral agents, were selected and assayed. These compounds were applied to verify the inhibitory activity for the protein function of 3CLpros (main proteases) of SARS-CoV and SARS-CoV-2. Among them, viral reverse-transcriptase inhibitors abacavir and tenofovir revealed a good inhibitory effect on both 3CLpros. Intriguingly, sildenafil, a cGMP-specific phosphodiesterase type 5 inhibitor also showed significant inhibitory function against them. The in silico docking study suggests that the active-site residues located in the S1 and S2 sites play key roles in the interactions with the inhibitors. The result indicates that 3CLpros are promising targets to cope with SAR-CoV-2 and its variants. The information can be helpful to design treatments to cure patients with COVID-19.

## 1. Introduction

Severe acute respiratory syndrome coronavirus 2 (SARS-CoV-2), with the first cases emerging from Wuhan, China [1], is currently spreading all over the world [2]. More than one hundred twenty million people have been infected, and over two million have died as of March 1, 2021. Regardless of the fatality of coronavirus disease 2019 (COVID-19), its impact and fear already surpassed the previous Severe Acute Respiratory Syndrome (SARS-CoV) of 2003 and Middle East Respiratory Syndrome (MERS) of 2012. A wide spectrum of illnesses, from mild-to-severe illness, including death [3,4,5], has been reported. The incubation period of 5–6 days (range 2–14 days) and the reproduction rate of 2.2–3.6 days of SARS-CoV-2 accelerate the spreading of COVID-19 [6,7]. Although there are several FDA-approved vaccines for SARS-CoV-2, there is no effective agent to cure COVID-19. Though many viral drugs, such as favipiravir (Avigan) [8], cidoforvir [9], abacavir [10] and lopinavir [11]; targeting enzymes against RNA-dependent RNA polymerase; viral DNA-polymerase; nucleoside reverse transcriptase; and viral protease have been developed, respectively, nothing is proven to treat COVID-19. Remdesivir is the only FDA-approved agent; however, its single-use form does not fully relieve symptoms. Since currently available vaccines are not perfect to protect and cure people from COVID-19, all kinds of scientific strategies are required to cope with the crisis through developing anti-SARS-CoV treatments, too.

Viral proteases have been studied and developed to produce antiviral agents against various viruses such as human immunodeficiency viruses (HIV), hepatitis C virus (HCV) and Human Rhinovirus 3C Protease. There are nine and two FDA-approved medicines targeting HIV and HCV proteases [12], respectively. Therefore, viral proteases are good targets to cope with viral diseases. In the case of coronaviruses, SARS-CoV or SARS-CoV-2, their viral proteases have been extensively studied to develop anti-SARS-CoV-2 treatments. With the efforts, several X-ray crystal structures of the main protease (3CLpro or Mpro) of SARS-CoV-2 complexed with inhibitors have been reported [13,14,15]. The crystal structures are quite beneficial to computer scientists who calculate the feasibility of interactions between 3CLpro and chemicals. Specifically, virtual screening of 3CLpro with FDA-approved drug databases as a drug-repurposing strategy [16] can speed up the search for promising drug candidates against COVID-19.

Nowadays, many variants of SARS-CoV-2s are appearing with altered spike proteins, which can nullify the effectiveness of vaccines (https://www.gisaid.org/, accessed on 1 March 2021). Therefore, enzymes can be alternative antiviral agent targets because mutations of their active sites are rare, due to their essential functions to sustain viral pathogenicity. Therefore, SARS-CoVs-2 3CLpro was considered and selected as a good COVID-19 target in this study because its activity is essential for viral processing. The previous study already showed that 3CLpros of SARS-CoV and SARS-CoV-2 are inhibited by the same flavonoids, indicating that both 3CLpros are structurally quite similar to each other, as shown in 94% sequence identity [17].

In this study, 3CLpros from SARS-CoV and SARS-CoV-2 were screened to find inhibitory compounds, using various commercially available antiviral drugs currently disputed on their effectiveness on COVID-19. Some of them have been currently applied and surveyed to monitor and cure patients with the symptoms of COVID-19. The result obtained in this study may be useful to those who consider these drugs as potential anti-COVID-19 agents.

## 2. Materials and Methods

### 2.1. Protein Expression and Purification of SARS-CoV 3CLpros

The coding sequences of SARS-CoV 3C-like proteinase (NCBI Reference sequence NP_828863.1) and SARS-CoV-2 3C-like proteinase (NCBI Reference sequence YP_009725301.1) were synthesized chemically by Bioneer (Daejeon, Korea) and cloned into a bacteriophage T7-based expression vector, respectively. The plasmid DNA was transformed into *E. coli* BL21 (DE3) for protein expression. *E. coli* BL21 (DE3) cells were grown on Luria–Bertani (LB) agar plates containing 150 μg mL^−1^ ampicillin. Several colonies were picked and grown in capped test tubes with 10 mL LB broth containing 150 μg mL^−1^ ampicillin. A cell stock composed of 0.85 mL culture and 0.15 mL glycerol was prepared and frozen at 193 K, for use in a large culture. The frozen cell stock was grown in 5 mL LB medium and diluted into 1000 mL fresh LB medium. The culture was incubated at 310 K, with shaking, until an OD_600_ of 0.6–0.8 was reached. At this point, the expression of SARS-CoV-2 3CLpros was induced by using isopropyl-β-d-1-thiogalactopyranoside (IPTG) at a final concentration of 1 mM. The culture was further grown at 310 K for 3 h, in a shaking incubator. Cells were harvested by centrifugation at 7650× *g* (6500 rev min^−1^), for 10 min, in a high-speed refrigerated centrifuge at 277 K. The cultured cell paste was resuspended in 25 mL of a buffer consisting of 20 mM Tris pH 7.5, 1 mM phenylmethylsulfonyl fluoride (PMSF) and 10 μg ml^−1^ DNase I. The cell suspension was disrupted by using an ultrasonic cell disruptor (Digital Sonifier 450, Branson, USA). Cell debris was pelleted by centrifugation at 24,900× *g* (15 000 rev min^−1^), for 30 min, in a high-speed refrigerated ultra-centrifuge at 277 K.

For SARS-CoV 3CLpro, the protein was purified by cation chromatography using a 5 mL Hi-Trap Q column (GE Healthcare, Piscataway, NJ, USA). The column was equilibrated with a buffer consisting of 20 mM MES pH 8.0, and the pooled fractions were loaded. The column was eluted by using a linear NaCl gradient to 1.0 M NaCl, and the protein was eluted at 0.28 M NaCl. The protein was concentrated in a buffer consisting of 0.28 M NaCl and 20 mM MES pH 8.0.

For SARS-CoV-2 3CLpro, the protein was purified by cation chromatography using a 5 mL Hi-Trap Q column (GE Healthcare, Piscataway, NJ, USA). The column was equilibrated with a buffer consisting of 20 mM Tris pH 7.5 and the pooled fractions were loaded. The column was eluted using a linear NaCl gradient to 1.0 M NaCl and the protein was eluted at 0.18 M NaCl. The purified protein was buffer exchanged into 20 mM Bis-Tris pH 7.5 using Vivaspin 20 MWCO 10 kDa (GE Healthcare), a centrifugal device. SDS–PAGE showed one band around 34 kDa (33796.64 Da), corresponding to the molecular weight of SARS-CoV-2 3CLpro (Supplementary Figure S1).

### 2.2. FRET Protease Assays with SARS-CoV 3CLpros

The custom-synthesized fluorogenic substrate, DABCYL-KTSAVLQSGFRKME-EDANS (ANYGEN, Gwangju, Korea), was used as a substrate for the proteolytic assay using SARS-CoV 3CLpro and SARS-CoV-2 3CLpro [18]. This substrate contains the nsp4/nsp5 cleavage sequence, GVLQ↓SG [19], and works as a generic peptide substrate for many coronaviruses including the SARS-CoV-2 3CLpro. The peptide was dissolved in distilled water and incubated with each protease. A SpectraMax i3x multi-mode microplate reader (Molecular Devices) was used to measure spectral-based fluorescence. The proteolytic activity was determined at 310 K by following the increase in fluorescence (λ_excitation_ = 340 nm, λ_emission_ = 490 nm, bandwidths = 9, 15 nm, respectively) of EDANS upon peptide hydrolysis as a function of time. Assays were conducted in black 96-well plates (Nunc) with 300 μL assay buffers containing protease and substrate, as follows: For the 3CLpros assay, the final concentration of the protease, peptide and chemical used at the assay was 1, 2.5 and 80 μM, each at 310 K, before measuring Relative Fluorescence Units (RFU). The reaction time was 2 h and 30 min for SARS-CoV 3CLpro, and 5 h for SARS-CoV-2 3CLpro. Before the assay, the emission spectra of antiviral agents and some of their adjuvants were surveyed after illuminating at 340 nm, to avoid overlapping with the emission spectrum of EDANS. Every compound was suitable to be tested. At first, the 3CLpro and chemical were mixed and preincubated at room temperature for 1 h. The reaction was initiated by the addition of the substrate, and each well was incubated at 310 K for the appropriate reaction time. After that, we measured the fluorescence of the mixture on the black 96-well plate, using the endpoint mode of SpectraMax i3x, where the excitation wavelength was fixed to 340 nm, and the emission wavelength was set to 490 nm, using 9 and 15 nm bandwidth, respectively. All reactions were carried out in triplicate. Among the twenty-four chemicals (Supplementary Table S1), five of them were picked up to further assay at a concentration range of 2~320 μM. The IC_50_ value, which is the value causing 50% inhibition of SARS-CoV 3CLpros, was calculated by nonlinear regression analysis, using GraphPad Prism 7.03 (GraphPad Software, San Diego, CA, USA).

### 2.3. FRET Protease Assays with SARS-CoV 3CLpros in the Presence of Triton X-100

The proteolytic assay using SARS-CoV 3CLpros in the presence of Triton X-100 was performed to differentiate the artificial inhibitory activity of chemicals through non-specific binding with proteases by forming aggregate or complexation. The concentration used in this study was 0.01%.

### 2.4. Ligand Preparation, Target Preparation and Induced-Fit Docking

All the docking and scoring calculations were performed using the Schrödinger software suite (Maestro, version 11.8.012). The compounds were extracted from the PubChem database in the SDF format and were combined in one file. The file was then imported into Maestro and prepared for docking, using LigPrep. The atomic coordinates of the crystal structure of SARS-CoV 3CLpro (4WY3) and SARS-CoV-2 3CLpro (7LOD) were retrieved from the Protein Data Bank and prepared by removing all solvent and adding hydrogens and minimal minimization in the presence of bound ligand, using Protein Preparation Wizard. Ionizer was used to generate an ionized state of all compounds at the target pH 7.0  ±  2.0. This prepared low-energy conformers of the ligand were taken as the input for an induced-fit docking. The induced-fit docking protocol [20] was run from the graphical user interface accessible within Maestro 11.8.012. Receptor sampling and refinement were performed on residues within 5.0 Å of each ligand for each of the ligand-protein complexes. With Prime [21], a side-chain sampling and prediction module, as well as the backbone of the target protein, were energy minimized. A total of induced-fit receptor conformations were generated for each of the ligands. Re-docking was performed with the test ligands into their respective structures that are within 30.0 kcal/mol of their lowest energy structure. Finally, the ligand poses were scored by using a combination of Prime and GlideScore scoring functions [22].

## 3. Results & Discussion

The severe expansion of COVID-19 caused by SARS-CoV-2 has urgently requested the need for new medicines or therapeutic alternatives to alleviate and stop symptoms of infected patients. However, there is no drug developed only for focusing on curing COVID-19 until now. Therefore, the study to use therapeutic alternatives provides a promising hope at this moment and many research groups have adopted drug repurposing approaches. For example, remdesivir designed to treat Ebola virus infections and lopinavir/ritonavir targeting the human immunodeficiency virus type 1 (HIV-1) protease have been investigated. Chloroquine and hydroxychloroquine are also applied in this approach. Clinical trials with various therapeutic alternatives are going on worldwide.

**Figure 1:**
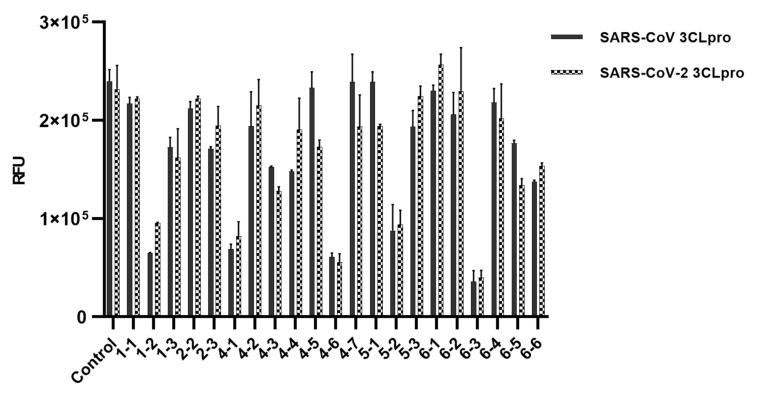
Inhibitory screening data of the chemical library against SARS-CoV and SARS-CoV-2 3CLpros. All chemical (40 μM) were confirmed for their inhibitory potential through a comparison of actual absorbance with control at 490 nm. Three chemicals (Nos. 2-1, 3-1 and 5-4) were not plotted, due to the abnormal surge of Relative Fluorescence Units (RFU) after the proteolytic cleavage of the substrate. The RFU are plotted against the log-concentration of inhibitory compounds. Each dot is expressed as the mean ± standard error of the mean (n = 3).

**Figure 2:**
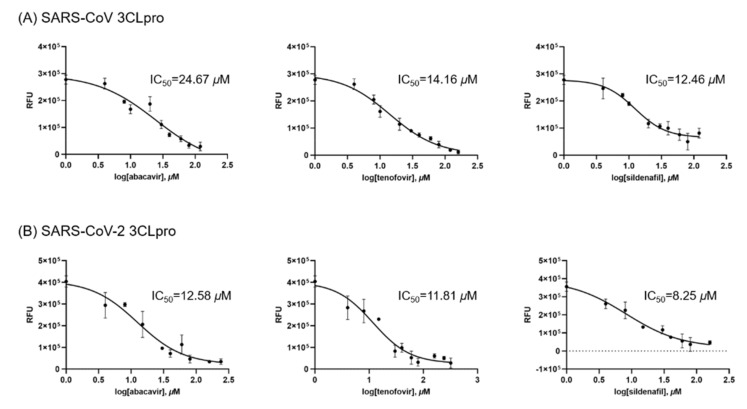
Results from the FRET method. Each data point represents the effect of each inhibitory compound against (**A**) SARS-CoV 3CLpro and (**B**) SARS-CoV-2 3CLpro, compared to the control. The RFU are plotted against the log-concentration of inhibitory compounds. Each dot is expressed as the mean ± standard error of the mean (n = 3). RFU = Relative Fluorescence Units.

In order to show 3CLpros are good targets to cure COVID-19, two 3CLpros were assayed and compared. A chemical library composed of twenty-four chemicals was built up (Table 1). These compounds had been used in various viral and other diseases alone or combined. They also have been applied to find SARS-CoV-2 treatments through virtual screenings or drug repurposing strategies [23,24,25,26,27,28,29,30,31,32,33,34,35,36,37,38,39,40,41,42]. The library contains antiviral agents of three DNA-polymerase inhibitors, three RNA polymerase inhibitors, one integrase, seven reverse transcriptase inhibitors, and four protease inhibitors. Six other chemicals include two antimalarial agents, two cGMP-specific phosphodiesterase type 5 inhibitors (sildenafil and tadalafil), cytochrome P450–3A (CYP3A) inhibitor (cobicistat), and CYP3A4 inhibitor (ritonavir). We applied the library to assay two SARS-CoV 3CLpros. Using 24 chemicals, an inhibitory effect of each compound at 40 μM was tested. Approximately ten indicated no activity, and six (No. 1-3, 2-3, 4-3, 4-4, 6-5, 6-6) little and five good activities. Their overall inhibitory patterns against two SARS-CoV 3CLpros were similar to each other (Figure 1). For evaluating their inhibitory potency, IC_50_ values of five compounds were determined. Intriguingly, two reverse transcriptases, abacavir and tenofovir, and one other chemical, sildenafil were found to have good inhibitory activity. The binding affinity data were plotted as log inhibitor concentration versus percent fluorescence inhibition (Figure 2).

The assay result indicated several interesting points for some viral agents currently applied in clinical trials in patients with COVID-19. At first, cidofovir, a DNA polymerase inhibitor, possesses a little activity with IC_50_ values was over 47 and 36 μM for SARS-CoV 3CLpro and SARS-CoV-2 3CLpro, respectively. However, its efficacy seems not promising. Second, abacavir and tenofovir have a substantial effect inhibitory activity with IC_50_ values of 24.67 and 14.16 μM for SARS-CoV 3CLpro and 12.58 and 11.81 μM for SARS-CoV-2 3CLpro, respectively. It is quite an interesting result in that they were originally designed to inhibit HIV reverse transcriptase. Third, three protease inhibitors including (Lopinavir/ritonavir) did not block the proteolytic activity of SARS-CoV-2 3CLpro. However, indinavir, an HIV protease inhibitor, revealed good inhibitory activity with an IC_50_ value of 31.45 and 13.61 μM for SARS-CoV 3CLpro and SARS-CoV-2 3CLpro, respectively. At last, sildenafil displayed a significant inhibitory activity in contrast to tadalafil. Its IC_50_ value is 12.46 and 8.247 μM for SARS-CoV 3CLpro and SARS-CoV-2 3CLpro, respectively. It is well-known that a strong anti-inflammatory effect of sildenafil [43,44] is the key function expected to relieve the symptom of COVID-19. The usage of sildenafil may expect additional merit due to its inhibitory activity against SARS-CoV-2 3CLpros as shown in this study.

SARS-CoV and SARS-CoV-2 3CLpros share 96% sequence identity. At the active site pockets of SARS-CoV-2 3CLpro, only two amino acids, Ser46 and Val86, are substituted by Ala46 and Leu86, respectively, in the case of SARS-CoV. However, Ala46 and Leu86 locate 10 Å and 14 Å away from Cys145, respectively, and thus their influence seems to be weak. This study showed that SARS-CoV and SARS-CoV-2 3CLpros have been inhibited similarly with several viral drugs. It implies that the extent of variance among 3CLpros at the structural level is quite limited. Since there are many subtypes of SARS-CoV-2 that have been reported [45], SARS-CoV-2 3CLpros are good targets to develop agents to cure COVID-19 caused by SARS-CoV-2 variants.

**Figure 3:**
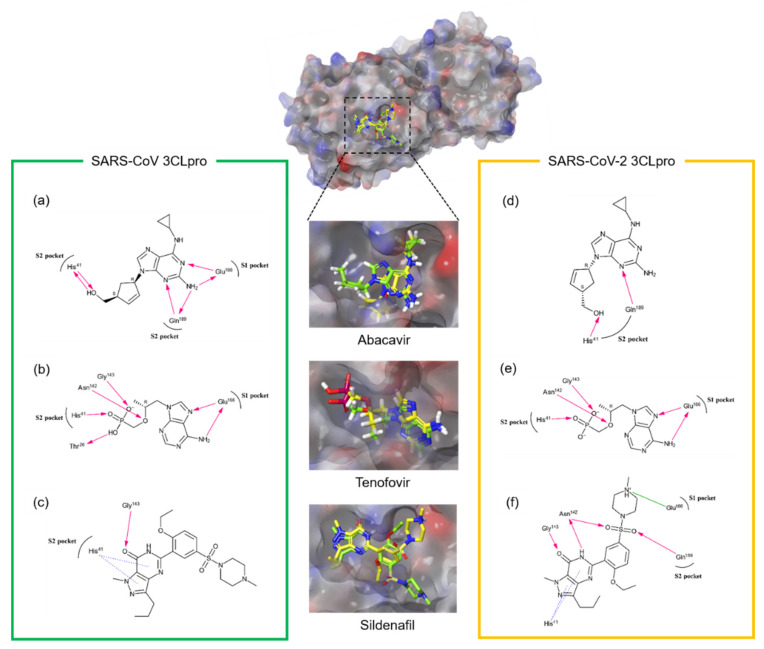
Predicted docking modes of each inhibitory compound in the catalytic site of SARS-CoV CLpro and SARS-CoV-2 CLpro. Docking poses of (**a**,**d**) abacavir, (**b**,**e**) tenofovir and (**c**,**f**) sildenafil were overlapped and depicted on the semi-transparent electrostatic surface potentials (red, negative; blue, positive; white, uncharged) of SARS-CoV (green) and SARS-CoV-2 (yellow) 3CLpros. The 2D schematic representation of each inhibitory compound was also drawn. Figures were created with Maestro v11.5.011. S1 represents the polar S1 site of 3CLpros; S2 for the hydrophobic S2 site. The pink arrows represent hydrogen bond interaction, the blue dot line is for π–π stacking and the green line is for salt bridge.

An in silico docking study on the most prominent three chemicals, abacavir, tenofovir and sildenafil has been done to deduce their binding mode and binding affinity. The glide scores of three compounds obtained against SARS-CoV 3CLpro were −8.517, −7.657 and −8.663, and those for SARS-CoV-2 3CLpro were −7.655, −7.142 and −8.405, respectively. The binding mode of each compound against both 3CLpros was also similar to each other (Figure 3).

In the case of abacavir on SARS-CoV-2 3CLpro, the 3-nitrogen atom of the purine moiety and the methanol moiety are critical to binding with the catalytic site through Gln189 and His41, respectively Additional interactions were observed in the case of SARS-CoV 3CLpro. The 2-amino group and 1-nitrogen atoms of the purine moiety also participated through Glu166 and Gln189 (Figure 3a,d).

Tenofovir interacts with Glu166 of SARS-CoV-2 3CLpro through the 6-amino group and the 7-nitrogen atom of the purine moiety. Gly143, Asn142 and His41 also bind to the phosphonic acid group. In the case of SARS-CoV 3CLpro, one additional hydrogen bond was also present between Thr28 and the phosphonic group (Figure 3b,e).

In the case of sildenafil, the two π–π stacking interactions between the pyrazolopyrimidine moiety and His41 of both 3CLpors are important. Gly143 also participates in the hydrogen bond to the oxo group. The interaction of Gln189 and Glu166 with the sulfonyl and methylpiperazine moiety, respectively, are also present in the case of SARS-CoV-2 3CLpro (Figure 3c,f). The docking results indicate that three compounds occupied both S1 and S2 sites of 3CLpros. The previously reported active site residues (His41, Gly143, Asn142, Glu166 and Gln189) predicted to interact with flavonoids [17] also play a key role in these cases.

There have been published several in silico docking studies targeting SARS-CoV-2 3CLpro. Bharadwaj et al., 2020 [41] provided a docking result of tadalafil complexed with the crystal structure of 3Clpro (6LU7) with AutoDock Vina. The paper predicted strong interactions of tadalafil with active site residues of 3CLpro including His41, Gly143, Asn142, Glu166 and Gln189. Qiao et al. (2021) [40] also reported their calculation on 2454 FDA-approved drugs docked on the same crystal structure with several AutoDock programs. Tadalafil was ranked in second place. Unfortunately, tadalafil did not display meaningful inhibitory activity against both 3CLpros in our experiment (Figure 1). In contrast, sildenafil, ranked in fourth place, was proved to possess a good 3CLpro inhibitor activity in this study. Beck et al. (2020) [27] applied a similar approach with 3410 FDA-approved drugs and suggested remdesivir and ritonavir as potential inhibitors against 3CLpro. However, they also did not show clear inhibitory activity, though remdesivir displayed a minor inhibitory activity (data not shown). Indu et al. (2020) [30] suggested raltegravir (top) and indinavir (third) as inhibitory candidates out of 65 FDA-approved small-molecule antiviral drugs. Our assay result showed a moderate inhibitory activity for that indinavir (Figure 1).

In this study, three compounds (abacavir, tenofovir and sildenafil) turned out to be effective for both 3CLpros. Two compounds (cidofovir and indinavir) also possessed inhibitory activity. Intriguingly, indinavir and sildenafil were predicted to block SARS-CoV-2 3CLpro activity by in silico docking studies [27,40]. Therefore, if the drug repurposing technique is well aligned with the computational approach, the success rate of finding potential drug candidates seems to be severely increased. The chemicals found in this study can be used to develop solely for SARS-CoV-2 3CLpro inhibitors or dual-target treatments against SARS-CoV-2 3CLpro and reverse transcriptase, for an example. Thus, these compounds can be used as good templates to develop better anti-SARS-CoV-2 agents.

## 4. Conclusions

The recent pandemic caused by SARS-CoV-2 is going on severely and thus patients with COVID-19 are exponentially increasing. In this study, some of the medicines currently used in clinical trials in patients with COVID-19 were investigated for their interactions with SARS-CoV-2 3CLpro. Among them, three compounds, abacavir, tenofovir and sildenafil, turned out to block the proteolytic function of 3CLpros from SARS-CoV and SARS-CoV-2. Among them, tenofovir has been known to kill SARS-CoV-2 in infected Vero cells [46]. It is very obvious that none of them play a major role as a SARS-CoV-2 3CLpro blocker until now. However, this study suggests that 3CLpro is a good target to design anti-SARS-CoV-2 drugs. Besides this, the information of the compounds displaying SARS-CoV-2 3CLpro inhibitory activity can be applied to design treatments against to cure patients with COVID-19. If other studies with RNA-dependent RNA polymerase, papain-like protease, etc., from SARS-CoV-2 follow the combined information from molecular biology, bioinformatics, pharmaceutical science and medicinal chemistry, it may lead to finding a strategy to save the lives of patients with COVID-19.

## Figures and Tables

**Table 1:** A chemical library targeting viral enzymes.

No	Name of Compound	Molecular Weight	Molecular Formula	CAS Number	Reference
1-1	Acyclovir	225.20	C_8_H_11_N_5_O_3_	59277-89-3	[23]
1-2	Cidofovir	279.19	C_8_H_14_N_3_O_6_P xH_2_O	113852-37-2	[24]
1-3	Ganciclovir	255.23	C_9_H_13_N_5_O_4_	82410-32-0	[25]
2-1	Favipiravir	157.10	C_5_H_4_FN_3_O_2_	259793-96-9	[26]
2-2	Remdesivir	291.27	C_12_H_13_N_5_O_4_	1809249-37-3	[27,28]
2-3	Sofosbuvir	529.45	C_22_H_29_FN_3_O_9_P	82-93-9	[29]
3-1	Raltegravir	482.51	C_20_H_20_FKN6O_5_	871038-72-1	[30]
4-1	Abacavir	225.20	C_8_H_11_N_5_O_3_	188062-50-2	[31]
4-2	Emtricitabine	247.25	C_8_H_10_FN_3_O_3_S	143491-57-0	[32]
4-3	Entecavir	277.28	C_12_H_15_N_5_O_3_	142217-69-4	[33]
4-4	Lamivudine	229.26	C_8_H_11_N_3_O_3_S	134678-17-4	[31]
4-5	Ribavirin	244.20	C_8_H_12_N_4_O_5_	36791-04-5	[31]
4-6	Tenofovir	305.23	C_9_H_14_N_5_O_4_P H_2_O	206184-49-8	[32]
4-7	Zidovudine	267.24	C_10_H_13_N_5_O_4_	30516-87-1	[34]
5-1	Darunavir	547.66	C_27_H_37_N_3_O_7_S	206361-99-1	[35]
5-2	Indinavir	613.79	C_36_H_49_N_5_O_8_S	180683-37-8	[30]
5-3	Lopinavir	628.80	C_37_H_48_N_4_O_5_	192725-17-0	[36]
5-4	Nafamostat mesylate	539.58	C_19_H_17_N_5_O_2_ 2CH_4_O_3_S	82956-11-4	[37]
6-1	Cobicistat	776.0	C_40_H_53_N_7_O_5_S_2_	1004316-88-4	[38]
6-2	Ritonavir	720.94	C_37_H_48_N_6_O_5_S_2_	155213-67-5	[27,39]
6-3	Sildenafil	666.70	C_22_H_30_N_6_O_4_S C_6_H_8_O_7_	171599-83-0	[40]
6-4	Tadalafil	389.40	C_22_H_19_N_3_O_4_	171596-29-5	[41]
6-5	Chloroquine	515.86	C_18_H_26_ClN_3_ 2H_3_PO_4_	50-63-5	[42]
6-6	Hydroxychloroquine	433.95	C_18_H_26_ClN_3_O H_2_SO_4_	747-36-4	[42]

DNA polymerase inhibitors, 1-1~3; RNA polymerase inhibitors, 2-1~3; integrase inhibitors, 3-1; reverse transcriptase inhibitors, 4-1~7; protease inhibitors, 5-1~4; others, 6-1~4.

## Data Availability

The data presented in this study are available on request from the corresponding author.

## Supplementary Materials

The following are available online, at https://www.mdpi.com/article/10.3390/microorganisms9040756/s1. Figure S1: The purification of SARS-CoV-2 3CLpro.

## References

[1] Zhu N., Zhang D., Wang W., Li X., Yang B., Song J., Zhao X., Huang B., Shi W., Lu R. (2020). China Novel Coronavirus Investigating and Research Team. A novel coronavirus from patients with pneumonia in China, 2019. N. Engl. J. Med..

[2] Olsen S.J., Chen M.Y., Liu Y.L., Witschi M., Ardoin A., Calba C., Mathieu P., Masserey V., Maraglino F., Marro S. (2020). Early introduction of severe acute respiratory syndrome coronavirus 2 into Europe. Emerg Infect. Dis..

[3] Chen N., Zhou M., Dong X., Qu J., Gong F., Han Y., Qiu Y., Wang J., Liu Y., Wei Y. (2020). Epidemiological and clinical characteristics of 99 cases of 2019 novel coronavirus pneumonia in Wuhan, China: A descriptive study. Lancet.

[4] Team TNCPERE (2020). The epidemiological characteristics of an outbreak of 2019 novel coronavirus diseases (COVID-19) in China. China CDC Wkly..

[5] Wu P., Hao X., Lau E.H., Wong J.Y., Leung K.S., Wu J.T., Cowling B.J., Leung G.M. (2020). Real-time tentative assessment of the epidemiological characteristics of novel coronavirus infections in Wuhan, China, as at 22 January 2020. Eurosurveillance.

[6] Biggerstaff M., Cauchemez S., Reed C., Gambhir M., Finelli L. (2014). Estimates of the reproduction number for seasonal, pandemic, and zoonotic influenza: A systematic review of the literature. BMC Infect. Dis..

[7] Zhao S., Lin Q., Ran J., Musa S.S., Yang G., Wang W., Lou Y., Gao D., Yang L., He D. (2020). Preliminary estimation of the basic reproduction number of novel coronavirus (2019-nCoV) in China, from 2019 to 2020: A data-driven analysis in the early phase of the outbreak. Int. J. Infect. Dis..

[8] Jin Z., Smith L.K., Rajwanshi V.K., Kim B., Deval J. (2013). The ambiguous base-pairing and high substrate efficiency of T-705 (Favipiravir) Ribofuranosyl 5’-triphosphate towards influenza a virus polymerase. PLoS ONE.

[9] Magee W.C., Hostetler K.Y., Evans D.H. (2005). Mechanism of inhibition of vaccinia virus DNA polymerase by cidofovir diphosphate. Antimicrob. Agents Chemother..

[10] Cruciani M., Martí-Carvajal A.J., Mengoli C., Serpelloni G., Bovo C., Moyle G. (2018). Abacavir versus other nucleoside reverse transcriptase inhibitor (NRTI) backbone therapies for treatment of HIV infection. Cochrane Database Syst. Rev..

[11] Sham H.L., Kempf D.J., Molla A., Marsh K.C., Kumar G.N., Chen C.M., Kati W., Stewart K., Lal R., Hsu A. (1998). ABT-378, a highly potent inhibitor of the human immunodeficiency virus protease. Antimicrob. Agents Chemother..

[12] Agbowuro A.A., Huston W.M., Gamble A.B., Tyndall J.D.A. (2018). Proteases and protease inhibitors in infectious diseases. Med. Res. Rev..

[13] Zhang L., Lin D., Sun X., Curth U., Drosten C., Sauerhering L., Becker S., Rox K., Hilgenfeld R. (2020). Crystal structure of SARSCoV-2 main protease provides a basis for design of improved α-ketoamide inhibitors. Science.

[14] Su H.X., Yao S., Zhao W.F., Li M.J., Liu J., Shang W.J., Xie H., Ke C.Q., Hu H.C., Gao M.N. (2020). Anti-SARS-CoV-2 activities in vitro of Shuanghuanglian preparations and bioactive ingredients. Acta Pharmacol. Sin..

[15] Fu L., Ye F., Feng Y., Yu F., Wang Q., Wu Y., Zhao C., Sun H., Huang B., Niu P. (2020). Both Boceprevir and GC376 efficaciously inhibit SARS-CoV-2 by targeting its main protease. Nat. Commun..

[16] Corsello S.M., Bittker J.A., Liu Z., Gould J., McCarren P., Hirschman J.E., Johnston S.E., Vrcic A., Wong B., Khan M. (2017). The Drug Repurposing Hub: A next-generation drug library and information resource. Nat. Med..

[17] Jo S., Kim S., Kim D.Y., Kim M., Shin D.H. (2020). Flavonoids with inhibitory activity against SARS-CoV-2 3CLpro. J. Enzym. Inhib. Med. Chem..

[18] Kuo C.-J., Chi Y.-H., Hsu J.T., Liang P.-H. (2004). Characterization of SARS main protease and inhibitor assay using a fluorogenic substrate. Biochem. Biophys. Res. Commun..

[19] Wu A., Wang Y.I., Zeng C., Huang X., Xu S., Su C., Wang M., Chen Y., Guo D. (2015). Prediction and biochemical analysis of putative cleavage sites of the 3C-like protease of Middle East respiratory syndrome coronavirus. Virus Res..

[20] Sherman W., Day T., Jacobson M.P., Friesner R.A., Farid R. (2006). Novel Procedure for Modeling Ligand/Receptor Induced Fit Effects. J. Med. Chem..

[21] Jacobson M.P., Pincus D.L., Rapp C.S., Day T.J., Honig B., Shaw D.E., Friesner R.A. (2004). A hierarchical approach to all-atom protein loop prediction. Proteins.

[22] Friesner R.A., Murphy R.B., Repasky M.P., Frye L.L., Greenwood J.R., Halgren T.A., Sanschagrin P.C., Mainz D.T. (2006). Extra precision glide: Docking and scoring incorporating a model of hydrophobic enclosure for protein-ligand complexes. J. Med. Chem..

[23] Kumar D., Kumari K., Bahadur I., Singh P. (2020). Promising Acyclovir and its derivatives to inhibit the protease of SARS-CoV-2: Molecular Docking and Molecular Dynamics simulations. Res. Sq..

[24] Jockusch S., Tao C., Li X., Anderson T.K., Chien M., Kumar S., Russo J.J., Kirchdoerfer R.N., Ju J. (2020). A library of nucleotide analogues terminate RNA synthesis catalyzed by polymerases of coronaviruses that cause SARS and COVID-19. Antivir. Res..

[25] Gurung A.B., Ali M.A., Lee J., Farah M.A., Al-Anazi K.M. (2020). Unravelling lead antiviral phytochemicals for the inhibition of SARS-CoV-2 Mpro enzyme through in silico approach. Life Sci..

[26] Shannon A., Selisko B., Huchting J., Touret F., Piorkowski G., Fattorini V., Ferron F., Decroly E., Meier C., Coutard B. (2020). Rapid incorporation of Favipiravir by the fast and permissive viral RNA polymerase complex results in SARS-CoV-2 lethal mutagenesis. Nat. Commun..

[27] Beck B.R., Shin B., Choi Y., Park S., Kang K. (2020). Predicting commercially available antiviral drugs that may act on the novel coronavirus (SARS-CoV-2) through a drug-target interaction deep learning model. Comput. Struct. Biotechnol. J..

[28] Pruijssers A.J., George A.S., Schäfer A., Leist S.R., Gralinksi L.E., Dinnon K.H. (2020). Remdesivir potently inhibits SARS-CoV-2 in human lung cells and chimeric SARS-CoV expressing the SARS-CoV-2 RNA polymerase in mice. bioRxiv.

[29] Jácome R., Campillo-Balderas J.A., de León S.P., Becerra A., Lazcano A. (2020). Sofosbuvir as a potential alternative to treat the SARS-CoV-2 epidemic. Sci. Rep..

[30] Indu P., Rameshkumar M.R., Arunagirinathan N., Al-Dhabi N.A., Valan Arasu M., Ignacimuthu S.R., Indinavir T. (2020). Dolutegravir, and Etravirine against main protease and RNA-dependent RNA polymerase of SARS-CoV-2: A molecular docking and drug repurposing approach. J. Infect. Public Health.

[31] Chien M., Anderson T.K., Jockusch S., Tao C., Li X., Kumar S., Russo J.J., Kirchdoerfer R.N., Ju J. (2020). Nucleotide Analogues as Inhibitors of SARS-CoV-2 Polymerase, a Key Drug Target for COVID-19. J. Proteome Res..

[32] Ayerdi O., Puerta T., Clavo P., Vera M., Ballesteros J., Fuentes M.E. (2020). Preventive Efficacy of Tenofovir/Emtricitabine Against Severe Acute Respiratory Syndrome Coronavirus 2 Among Pre-Exposure Prophylaxis Users. Open Forum Infect Dis..

[33] Peele K.A., Durthi C.P., Srihansa T., Krupanidhi S., Ayyagari V.S., Babu D.J. (2020). Molecular docking and dynamic simulations for antiviral compounds against SARS-CoV-2: A computational study. Inform. Med. Unlocked.

[34] Mostafa M.A. (2020). Role of Zidovudine and Candesartan in the Novel SARS-CoV-2 Treatment Trials; Theoretical Study. AIJR Prepr..

[35] Fintelman-Rodrigues N., Sacramento C.Q., Lima C.R., da Silva F.S., Ferreira A.C., Mattos M., de Freitas C.S., Soares V.C., Dias S.D.S.G., Temerozo J.R. (2020). Atazanavir, Alone or in Combination with Ritonavir, Inhibits SARS-CoV-2 Replication and Proinflammatory Cytokine Production. Antimicrob. Agents Chemother..

[36] Panagopoulos P., Petrakis V., Panopoulou M., Trypsianis G., Penlioglou T., Pnevmatikos I., Papazoglou D. (2020). Lopinavir/ritonavir as a third agent in the antiviral regimen for SARS-CoV-2 infection. J. Chemother..

[37] Hoffmann M., Schroeder S., Kleine-Weber H., Müller M.A., Drosten C., Pöhlmann S. (2020). Nafamostat Mesylate Blocks Activation of SARS-CoV-2: New Treatment Option for COVID-19. Antimicrob. Agents Chemother..

[38] Shytaj I.L., Fares M., Lucic B., Gallucci L., Tolba M.M., Zimmermann L., Ayoub A.T., Cortese M., Neufeldt C.J., Laketa V. (2021). The FDA-approved drug cobicistat synergizes with remdesivir to inhibit SARS-CoV-2 replication. bioRxiv.

[39] Choy K.T., Wong A.Y.L., Kaewpreedee P., Sia S.F., Chen D., Hui K.P.Y. (2020). Remdesivir, lopinavir, emetine, and homoharringtonine inhibit SARSCoV-2 replication in vitro. Antiviral. Res..

[40] Qiao Z., Zhang H., Ji H.-F., Chen Q. (2020). Computational View toward the Inhibition of SARS-CoV-2 Spike Glycoprotein and the 3CL Protease. Computation.

[41] Bharadwaj S., Azhar E.I., Kamal M.A., Bajrai L.H., Dubey A., Jha K., Yadava U., Kang S.G., Dwivedi V.D. (2020). SARS-CoV-2 Mpro inhibitors: Identification of anti-SARS-CoV-2 Mpro compounds from FDA approved drugs. J. Biomol. Struct. Dyn..

[42] Liu J., Cao R., Xu M., Wang X., Zhang H., Hu H., Li Y., Hu Z., Zhong W., Wang M. (2020). Hydroxychloroquine, a less toxic derivative of chloroquine, is effective in inhibiting SARS-CoV-2 infection in vitro. Cell Discov..

[43] Raposo C., Nunes A.K.D., Luna R.L.D., Araújo S.M.D., da Cruz-Höfling M.A., Peixoto C.A. (2013). Sildenafil (Viagra) Protective Effects on Neuroinflammation: The Role of iNOS/NO System in an Inflammatory Demyelination Model. Mediat. Inflamm..

[44] Nunes A.K.S., Rapôso C., Rocha S.W.S., de Sousa Barbosa K.P., de Almeida Luna R.L., da Cruz-Höfling M.A., Peixoto C.A. (2015). Involvement of AMPK, IKβα-NFκB and eNOS in the sildenafil anti-inflammatory mechanism in a demyelination model. Brain Res..

[45] Morais I.J., Polveiro R.C., Souza G.M., Bortolin D.I., Sassaki F.T., Lima A.T.M. (2020). The global population of SARS-CoV-2 is composed of six major subtypes. Sci. Rep..

[46] Clososki G.C., Soldi R.A., da Silva R.M., Guaratini T., Lopes J.N.C., Pereira P.R.R., Lopes J.L.C., dos Santos T., Martins R.B., Costa C.S. (2020). Tenofovir Disoproxil Fumarate: New Chemical Developments and Encouraging in vitro Biological Results for SARS-CoV-2. J. Braz. Chem. Soc..

