# Supplementary material for: A Study of 3CLpros as Promising Targets against SARS-CoV and SARS-CoV-2
Source: Microorganisms. 2021 Apr 3;9(4):756. doi: 10.3390/microorganisms9040756 (PMC8065850; doi:10.3390/microorganisms9040756)

A study of 3CLpros as promising targets against SARS-CoV and SARS-CoV-2

Seri Jo, Suwon Kim, Jahyun Yoo, Mi-Sun Kim and Dong Hae Shin*

College of Pharmacy and Graduates School of Pharmaceutical Sciences, Ewha W. University, Seoul, Republic of Korea 03760

Supplementary Figure 1. The purification of SARS-CoV-2 3CLpro

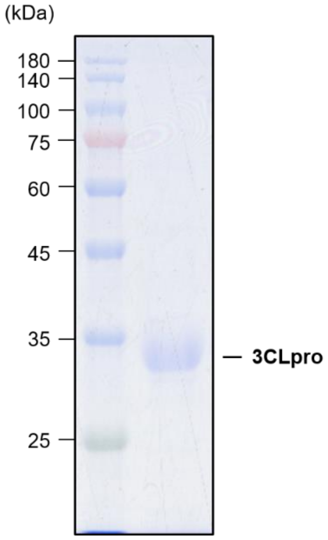

Supplement: Supplementary file 1 [file microorganisms-09-00756-s001.pdf]
